# Reduced exposure to malaria vectors following indoor residual spraying of pirimiphos-methyl in a high-burden district of rural Mozambique with high ownership of long-lasting insecticidal nets: entomological surveillance results from a cluster-randomized trial

**DOI:** 10.1186/s12936-021-03583-8

**Published:** 2021-01-21

**Authors:** Joseph M. Wagman, Kenyssony Varela, Rose Zulliger, Abuchahama Saifodine, Rodaly Muthoni, Stephen Magesa, Carlos Chaccour, Christelle Gogue, Kenzie Tynuv, Aklilu Seyoum, Dereje Dengela, Francisco Saúte, Jason H. Richardson, Christen Fornadel, Yvonne-Marie Linton, Laurence Slutsker, Baltazar Candrinho, Molly Robertson

**Affiliations:** 1 PATH, Washington, DC USA; 2 PMI VectorLink Project, Abt Associates, Maputo, Mozambique; 3 US President’s Malaria Initiative, US Centers for Disease Control and Prevention, Maputo, Mozambique; 4 US President’s Malaria Initiative, US Agency for International Development, Maputo, Mozambique; 5 Centro de Investigação Em Saúde de Manhiça, Maputo, Mozambique; 6 Barcelona Institute for Global Health, Barcelona, Spain; 7 PMI VectorLink Project, Abt Associates, Bethesda, MD USA; 8 VCC, Liverpool, UK; 9 Walter Reed Biosystematics Unit, Suitland, MD USA; 10 Smithsonian Institution—National Museum of Natural History, Washington, DC USA; 11 Programa Nacional de Controlo da Malária, Maputo, Mozambique

**Keywords:** Indoor residual spraying, 3GIRS, Pyrethroid resistance, *An. funestus*, Cluster-randomized trial

## Abstract

**Background:**

The need to develop new products and novel approaches for malaria vector control is recognized as a global health priority. One approach to meeting this need has been the development of new products for indoor residual spraying (IRS) with novel active ingredients for public health. While initial results showing the impact of several of these next-generation IRS products have been encouraging, questions remain about how to best deploy them for maximum impact. To help address these questions, a 2-year cluster-randomized controlled trial to measure the impact of IRS with a microencapsulated formulation of pirimiphos-methyl (PM) in an area with high ownership of long-lasting insecticidal nets (LLINs) was conducted in a high-transmission district of central Mozambique with pyrethroid resistant vectors. Presented here are the results of the vector surveillance component of the trial.

**Methods:**

The 2 year, two-armed trial was conducted in Mopeia District, Zambezia Province, Mozambique. In ten sentinel villages, five that received IRS with PM in October–November 2016 and again in October–November 2017 and five that received no IRS, indoor light trap collections and paired indoor-outdoor human landing collections catches (HLCs) were conducted monthly from September 2016 through October 2018. A universal coverage campaign in June 2017, just prior to the second spray round, distributed 131,540 standard alpha-cypermethrin LLINs across all study villages and increased overall net usage rates in children under 5 years old to over 90%.

**Results:**

The primary malaria vector during the trial was *Anopheles funestus *sensu lato (s.l.), and standard World Health Organization (WHO) tube tests with this population indicated variable but increasing resistance to pyrethroids (including alpha-cypermethrin, from > 85% mortality in 2017 to 7% mortality in 2018) and uniform susceptibility to PM (100% mortality in both years). Over the entire duration of the study, IRS reduced *An. funestus* s.l. densities by 48% (CI_95_ 33–59%; *p* < 0.001) in indoor light traps and by 74% (CI_95_ 38–90%; *p* = 0.010) during indoor and outdoor HLC, though in each study year reductions in vector density were consistently greatest in those months immediately following the IRS campaigns and waned over time. Overall there was no strong preference for *An. funestus* to feed indoors or outdoors, and these biting behaviours did not differ significantly across study arms: observed indoor-outdoor biting ratios were 1.10 (CI_95_ 1.00–1.21) in no-IRS villages and 0.88 (CI_95_ 0.67–1.15) in IRS villages. The impact of IRS was consistent in reducing HLC exposures both indoors (75% reduction: CI_95_ 47–88%; *p* = 0. < 0.001) and outdoors (68% reduction: CI_95_ 22–87%; *p* = 0.012). While substantially fewer *Anopheles gambiae* s.l. were collected during the study, trends show a similar impact of IRS on this key vector group as well, with a 33% (CI_95_ 7–53%; p = 0.019) reduction in mosquitoes collected in light traps and a non-statistically significant 39% reduction (p = 0.249) in HLC landing rates.

**Conclusion:**

IRS with PM used in addition to pyrethroid-only LLINs substantially reduced human exposures to malaria vectors during both years of the cluster-randomized controlled trial in Mopeia—a high-burden district where the primary vector, *An. funestus* s.l., was equally likely to feed indoors or outdoors and demonstrated increasing resistance to pyrethroids. Findings suggest that IRS with PM can provide effective vector control, including in some settings where pyrethroid-only ITNs are widely used.

*Trial registration* clinicaltrials.gov, NCT02910934. Registered 22 September 2016, https://www.clinicaltrials.gov/ct2/show/NCT02910934.

## Background

Vector control, primarily through the use of long-lasting insecticidal nets (LLINs) complemented with the indoor residual spraying (IRS) of insecticides, has contributed substantially to the overall reduction of malaria burden seen across Africa since 2000 [1, 2]. However, concerns are increasing that insecticide resistance in key malaria vector populations may be diminishing the efficacy of these two core vector control tools [3–5] and may be contributing to the overall plateau in progress toward malaria burden reduction goals reported recently [6, 7]. Of particular concern is resistance to pyrethroids, the most commonly used class of insecticide for IRS until around 2013 [8] and the only insecticide class recommended by the World Health Organization (WHO) for use in LLINs until 2018. As such, the need for new products and novel approaches to malaria vector control has been widely acknowledged as a top global health priority [9–11].

One approach to meeting this need has been the development of new products for IRS with novel active ingredients for public health: third-generation IRS (3GIRS) products that are defined as IRS products effective at killing pyrethroid-resistant mosquitoes and which have a target duration of residual efficacy lasting at least 6 months. The first of these products to receive WHO recommendation as a malaria vector control tool was a microencapsulated formulation of the organophosphate insecticide pirimiphos-methyl (PM): Actellic® 300CS (Syngenta AG, Basel, Switzerland). While initial results showing the impact of Actellic and other 3GIRS products have been encouraging [12–18], questions remain about where and how to best deploy them to maximize their impact.

To help address this knowledge gap, a cluster-randomized controlled trial designed to measure the impact of IRS with Actellic® 300CS used in addition to standard pyrethroid-only LLINs was recently completed in a high-transmission district in Zambezia Province, central Mozambique [19]. The present paper describes the impact of the intervention on local malaria vectors, providing valuable context to interpret the positive epidemiological effects observed [20] and helping to clarify some of the biological mechanisms that drive the public health impact of 3GIRS products in high-burden settings.

## Methods

### Study setting

The study setting, Mopeia District of Zambezia Province in central Mozambique, has been described in detail previously [19]. Briefly, Mopeia is a rural district with an estimated total population of 162,000 in 2016 [21]. Mozambique is one of the top five highest-risk countries in the world in terms of malaria burden, with an estimated 10 million cases in 2017 representing approximately 5% of the global burden of the disease [7]. Zambezia is among the highest-burden provinces in Mozambique, with a *Plasmodium falciparum* infection prevalence of 68% recorded in children 6–59 months old during the 2015 Malaria Indicator Survey [22]. Recent vector surveillance data from 2016 in Zambezia indicated that the most abundant malaria vector species group throughout the year is *Anopheles funestus *sensu lato (s.l.), though *Anopheles gambiae* s.l. is also present (primarily during the rainy season, from November–April) [23]. An additional consideration in choosing Mopeia as a study site was that in 2016 *An. gambiae* s.l. populations from the bordering districts of Morrumbala and Mocuba were shown to be resistant to pyrethroids (34% to 52% mortality 24 h after exposure to deltamethrin and 33% to 40% mortality 24 h after exposure to lambda-cyhalothrin using WHO diagnostic tube tests) [23].

Following a pre-study census enumeration in 2016, 194 villages in Mopeia District were organized into 168 distinct study clusters and randomized into one of two study arms: (1) the no-IRS arm, in which households continued to benefit from the standard National Malaria Control Programme (NMCP) vector control strategy of universal coverage with LLINs or (2) the IRS arm, in which households also received IRS with a microencapsulated formulation of PM (Actellic® 300CS) in October–November 2016 and again in October–November 2017, in addition to the standard LLIN strategy. All villages across both study arms also benefited from an NMCP-led mass LLIN campaign that distributed nets containing alpha-cypermethrin (MAGNet®, V.K.A. Polymers Pvt. Ltd.) in July 2017, as well as continuous routine distribution of LLINs during antenatal care and childhood vaccination program visits before and during the study. NMCP standard case management practices, which included free rapid diagnostic testing of all suspected cases passively reporting to the public health sector (either directly at a health facility or by consulting a community health worker) and provision of a full course of appropriate artemisinin-based combination therapy to all confirmed cases, were available to all district residents throughout the study.

**Fig. 1:**
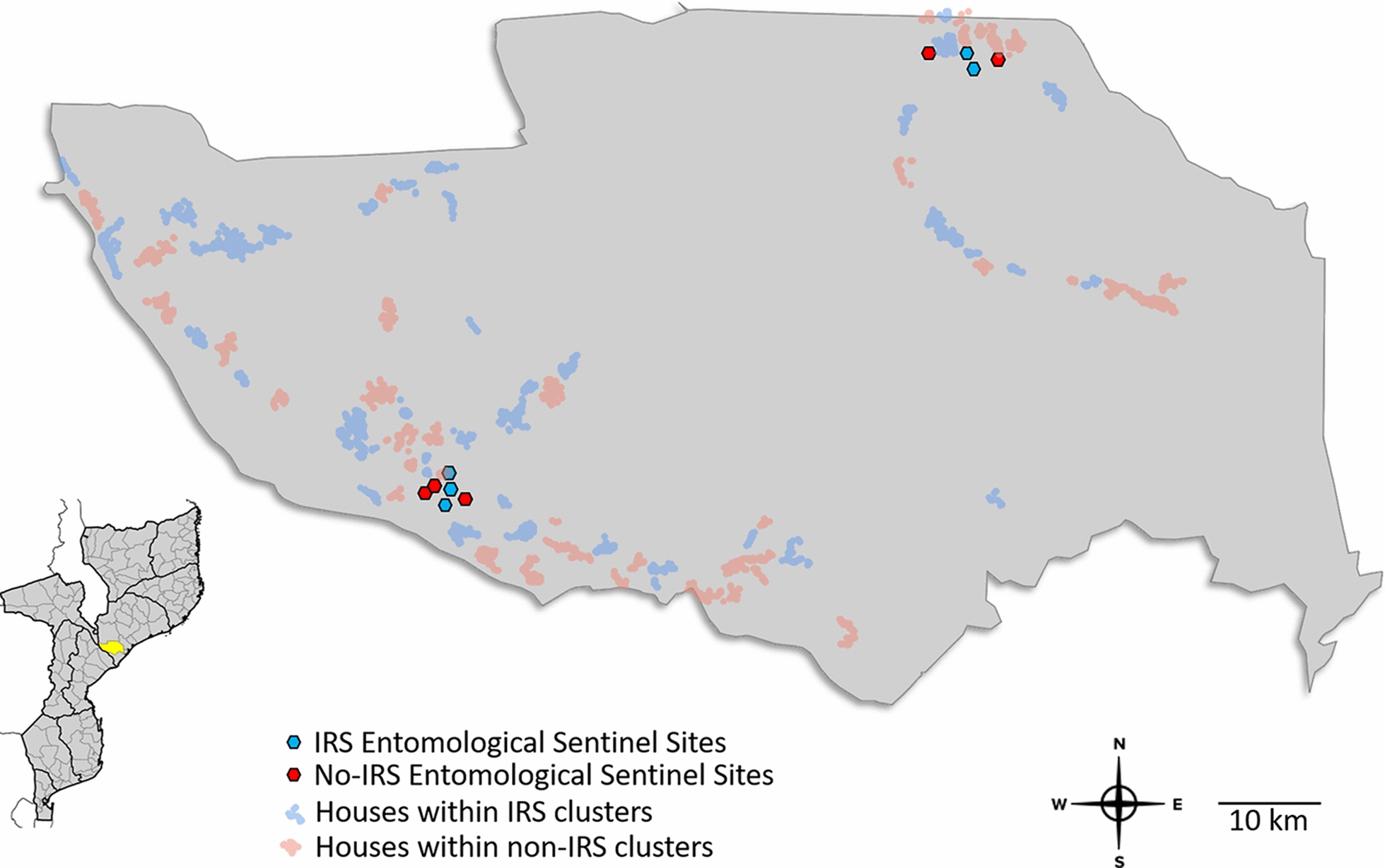
Locations of the entomological surveillance sites used during the cluster-randomized controlled trial of IRS with PM in Mopeia District, Mozambique: 2016 to 2018. In each village, indoor mosquito densities were sampled at eight sentinel houses for three consecutive nights every month (240 total trap-nights per month) using a human-baited CDC LT. In eight of the ten villages, paired indoor-outdoor HLCs were also conducted at one additional house for three consecutive nights every month (24 collection-nights per month). *CDC LT* US Centers for Disease Control light trap, *HLC* human landing collection, *IRS* indoor residual spraying, *PM* pirimiphos-methyl

Based on preliminary mosquito density surveys, ease of access, and safety, ten sentinel villages were selected for entomological surveillance activities during the cluster-randomized trial: five villages each from the IRS clusters and no-IRS clusters (Table 1; Fig. 1). In each village, eight sentinel houses were randomly selected from the core zone for longitudinal sampling with indoor light traps. In eight of the ten villages, an additional house was selected for longitudinal sampling by paired indoor-outdoor human landing catches (HLCs). IRS implementation and the entomological surveillance component of the study were conducted by the US President’s Malaria Initiative (PMI) and Abt Associates Inc. through the Africa Indoor Residual Spraying (AIRS) (September 2016–February 2018) and PMI VectorLink (March–October 2018) projects.

**Table 1:** The entomological surveillance sites used during the cluster-randomized controlled trial of IRS with PM in Mopeia District, Mozambique: 2016–2018

IRS clusters				No-IRS clusters			
Village	Village population^a^	Sentinel houses—CDC LT	Sentinel houses—HLC	Village	Village population	Sentinel houses—CDC LT	Sentinel houses—HLC
Eduardo Mondlane	4600	8	1	Zona Verde	3600	8	1
7 de Abril	2300	8	1	A Luta Continua	1500	8	1
4 de Outubro	2200	8	1	Lua Lua Sede	1300	8	–
25 de Junho	2300	8	1	Josina Machel	1000	8	1
Paz	700	8	–	Mirrongone	400	8	1

*CDC LT* US Centers for Disease Control light trap, *HLC* human landing collection, *IRS* indoor residual spraying, *PM* pirimiphos-methyl

^a^From 2016 pre-intervention census[18]

The insecticide susceptibility profile of local vector populations in the district were monitored in the study area using field-collected mosquitoes and standard WHO tube bioassays [24]. In 2017 and 2018, *An. funestus* s.l. adults were collected via Prokopack aspiration [25] from various collection points within Mopeia during the months of August and September, then tested against alpha-cypermethrin, deltamethrin, bendiocarb, dichloro-diphenyl-trichloroethane (DDT), and PM in 2017 [26] and against alpha-cypermethrin and PM in 2018 [27]. *Anopheles gambiae* s.l. larvae, also pooled from various collection points within Mopeia district, were reared to adults in the PMI AIRS insectary in Quelimane, Mozambique, and tested against alpha-cypermethrin and PM in both years [26, 27].

### IRS intervention

In IRS clusters, the PMI AIRS Mozambique team aimed to spray all eligible structures (defined by the PMI AIRS program as a free-standing structure in which people sleep/spend the night and has sprayable surfaces) with 1 g per square metre of PM, the target dose recommended by both the manufacturer and the WHO [28, 29]. The 2016 campaign lasted from October 10 until the first week of December, and 16,500 of 19,992 (83%) eligible structures encountered were sprayed. The 2017 campaign lasted from October 17 until December 13, during which 16,936 of 19,950 (85%) eligible structures encountered were sprayed [28, 29]. Quality control checks using standard WHO cone wall bioassays within 1 week of spray completion indicated high-quality IRS application in both years: adjusted 24-h mortality rates for susceptible *Anopheles arabiensis* (KGB strain) exposed to treated wall surfaces for 30 min ranged from 96.5 to 100% [28, 29]. Due to limited test mosquito availability, residual efficacy testing using the WHO cone wall bioassay was limited to 3 months after the 2016 campaign, but even then 24-h mortality was still greater than 95% [28]. Results in 2017 were similar, with 100% mortality observed at 3 months in all sites in Mopeia, and four-month mortality ranging from 35 to 82% [23, 26].

### LLIN ownership and use estimates

The July 2017 LLIN campaign distributed 131,540 standard alpha-cypermethrin LLINs across all villages in Mopeia. During a second, mop-up round of the LLIN campaign in November 2017, an additional 13,300 nets were distributed to fill coverage gaps, but no such gaps were identified in communities where entomological surveillance was conducted and they were therefore not included in this secondary campaign. Household LLIN ownership and LLIN use in the under-5-year-old population were estimated throughout the study during annual cross-sectional surveys and monthly home visits [19, 20]. Overall, the proportion of households that owned at least one LLIN, assessed during monthly cohort visits, increased from 61 to 63% before the mass distribution campaign to over 90% 2 months after the conclusion of the campaign. No differences were observed across study arms in the proportion of children under 5 years old who were reported to have slept under a net the night before monthly study-implemented household surveys, with estimates ranging from 59 to 67% before the mass distribution campaign and from 92 to 94% after the campaign [20].

### Entomological surveillance

#### Indoor light traps

US Centers for Disease Control miniature light traps (CDC LT) (John W. Hock Company, Gainesville, FL) were used to monitor indoor host-seeking mosquito densities in eight sentinel houses in each surveillance village. Traps were hung approximately 1.5 m from the ground at the foot of a bed with family members sleeping under an untreated bed net. Traps were operated overnight, from 1800 to 0600 h, on three consecutive nights every month, producing a total of 240 trap-nights every month (120 from houses in IRS clusters and 120 from houses in no-IRS clusters) from September 2016 through October 2018.

#### Human landing collections

In eight of the villages, an additional house was used for paired indoor-outdoor human landing collections (HLCs). HLCs were also conducted overnight from 1800 to 0600 h, with one collector seated outside (approximately 10 m from the entrance to the home) and one collector seated inside (approximately 5 m from the entrance to the home). Collectors sat quietly in a chair with legs exposed below the knee and collected mosquitoes that landed on them using a flashlight and mouth aspirator. During each hour, collectors collected mosquitoes for 50 min and rested for 10, during which they organized assigned collection cups; recorded the ambient temperature, humidity, and any rainfall; and exchanged indoor-outdoor positions. HLCs were conducted at each house for three consecutive nights every month, for a total of 24 paired collection-nights per month (12 from houses in IRS clusters and 12 from houses in no-IRS clusters).

#### Morphological and molecular characterization of mosquito specimens

All Anopheles mosquitoes collected were morphologically identified to species or species group, as best as possible, using the best-available comprehensive key [30]. A subsample of mosquitoes collected during HLC and CDC LT surveillance (24% of the total collected; 6216/26,348) were further analysed by the Walter Reed Biosystematics Unit, Suitland, MD. Though the goal was to subsample approximately 25% of all mosquitoes collected, specimen degradation during transport and storage ultimately resulted in an uneven subsampling distribution and not all collection months and locations were equally represented.

For molecular analysis, the head and thorax portion were separated from each individual mosquito and used for DNA extraction using the AutoGen® robotic extraction platform. Specimens morphologically identified as *An. gambiae* s.l. or *An. funestus* s.l. were verified using available species-diagnostic polymerase chain reaction (PCR) assays for the *An. gambiae* complex [31, 32] or *An. funestus* group [33, 34], as recommended by MR4 [35]. Where amplification was not achieved using the aforementioned assays, or original morphological identifications were discordant with these species groups, molecular identifications were verified by PCR amplification of the second internal transcribed spacer region of the nuclear rDNA (ITS2) and/or the barcoding region of the mtDNA cytochrome c oxidase I (COI) gene, according to previously described conditions [36]. Infection with *P. falciparum* or *Plasmodium vivax* was determined by screening the head-thorax extractions using a demultiplexed version of the *Plasmodium* detection assay [37], recommended by MR4 [35]. All resultant species-identification (COI/ITS2) and *Plasmodium* amplicons were bidirectionally sequenced using the original PCR primers on an ABI® 3733 sequencer in the Laboratories of Analytical Biology, Smithsonian Institution—National Museum of Natural History. Raw chromatograms were edited in Sequencher® v5.4.6 and the resultant consensus sequences compared with morphologically verified reference barcodes compiled by Walter Reed Biosystematics Unit and the Mosquito Barcoding Initiative in the BOLD database (www.boldsystems.org) and existing entries in GenBank (https://www.ncbi.nlm.nih.gov/genbank).

#### Estimates of entomological inoculation rates

Despite the poorly representative subsample of mosquitoes collected that provided valid parasite screening results, crude estimates of monthly entomological inoculation rates (EIRs) were calculated by multiplying monthly *An. funestus* s.l. sporozoite rates by the corresponding average number of *An. funestus* s.l. mosquitoes collected per CDC LT collection-night, per village.

#### Statistical analysis

Collection results were aggregated by month and summarized using Microsoft Excel® v1906 and Tableau® v2019.1. Crude differences in vector abundance across IRS and no-IRS surveillance sites are presented using an [ln(n + 1)] transformation to estimate the geometric mean number of mosquitoes collected by trap-night and associated 95% confidence intervals. To accommodate non-normal mosquito distributions and the high proportion of collection-nights in which no mosquitoes were collected, formal statistical analyses testing differences in mosquito density across study arms were performed using a zero-inflated negative binomial regression model ([zinb] in STATA® v14.2) with robust standard errors clustered at the village level to estimate incidence rate ratios (IRRs) comparing the rate at which mosquitoes were collected per location-night in IRS compared to no-IRS villages (as well as indoors compared to outdoors for the HLC results).

#### Ethical considerations

This research protocol was reviewed and approved by The National Bioethical Committee of Mozambique (Comité Nacional de bioética para a Saúde de Moçambique [CNBS]), the Institutional Review Board of CISM (CIBS-CISM), the PMI Operational Research Committee, and the Research Ethics Committee at PATH. The CDC reviewed the study as human subjects research with non-engagement by CDC staff. The study is registered at http://www.clinicaltrials.gov, under registration number NCT02910934.

## Results

### Vector density and species composition

**Fig. 2:**
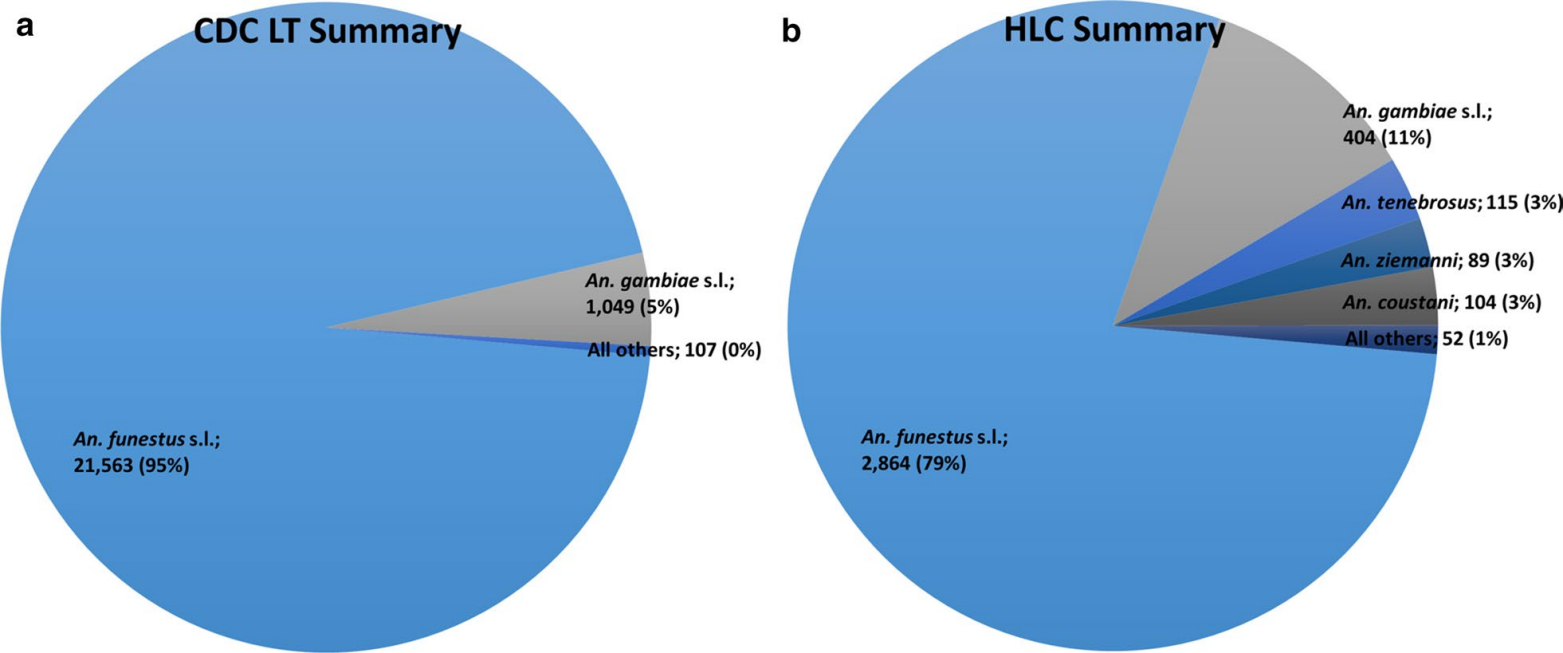
Overall densities and species group distributions for *Anopheles* mosquitoes collected **a** in CDC LTs and **b** with HLCs, during the cluster-randomized trial in Mozambique: 2016–2018. Monthly totals, grouped by village IRS status, for *An. funestus* s.l. and *An. gambiae* s.l. are presented in Tables 2, 3. All species group designations were based on morphological identifications in the field. *CDC LT* US Centers for Disease Control light trap, *HLC* human landing collection, *IRS* indoor residual spraying

**Fig. 3:**
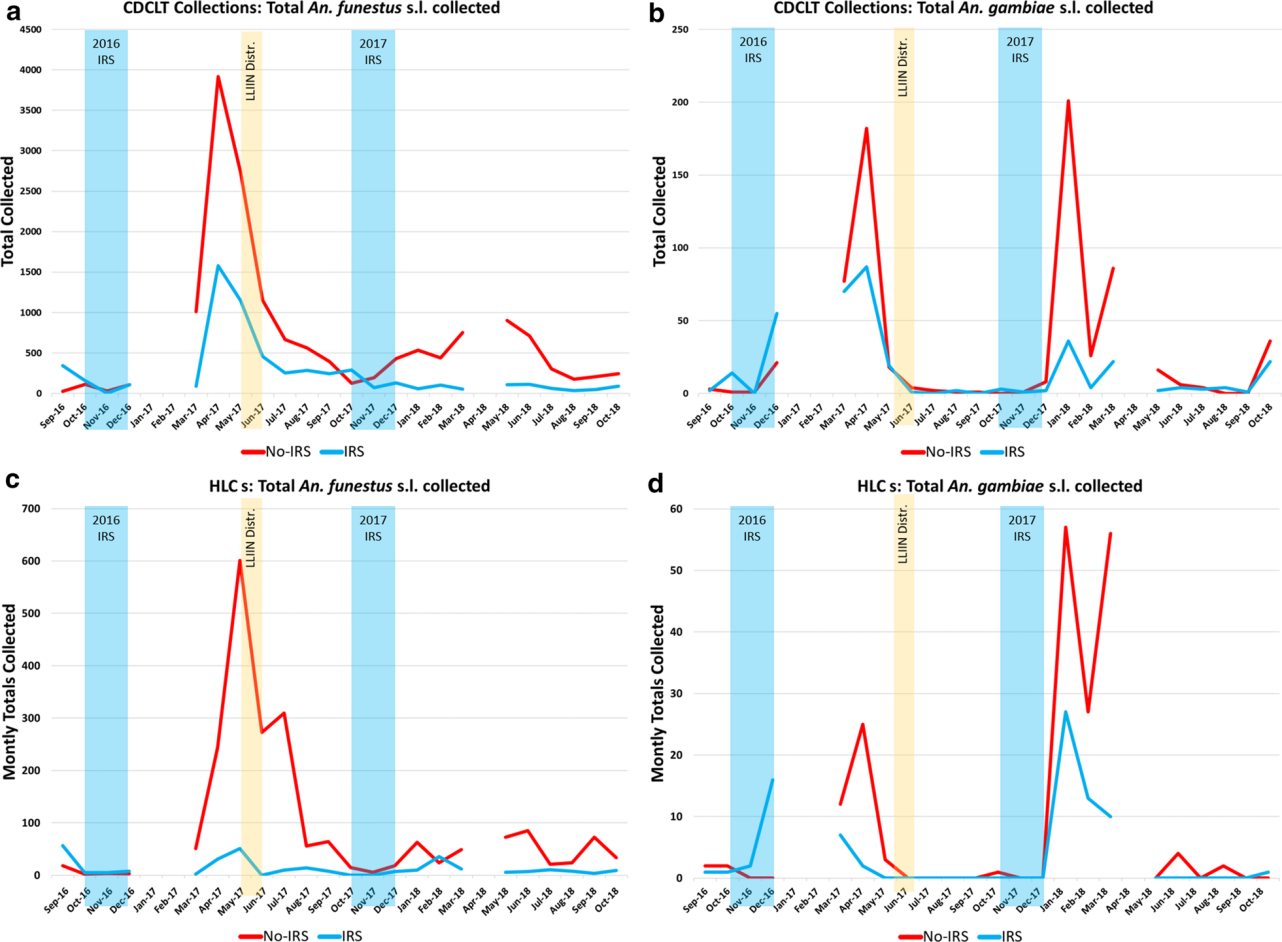
Monthly trends in total mosquitoes collected during the cluster-randomized trial using indoor, human-baited CDC LTs: **a** *An. funestus* s.l. and **b** *An. gambiae* s.l.; and paired indoor-outdoor HLCs: **c** *An. funestus* s.l. and **d** *An. gambiae* s.l. Each study arm had an equal number of collection nights per month. *CDC LT* US Centers for Disease Control light trap, *HLC* human landing collection

In total, 22,719 female *Anopheles* from nine morphologically identified species groups were collected in CDC LTs, and 3629 mosquitoes from 15 morphologically identified species groups were collected via HLC (Fig. 2; Additional file 1: Table S1) from September 2016 through October 2018. Data from 3 months are missing: specimens and data from January and February 2017 were lost in an insectary fire, and collections from April 2018 were not able to be performed. The most abundant taxon collected was *An. funestus* s.l., comprising 95% of all CDC LT specimens collected and 79% of all HLCs. The second most abundant was *An. gambiae* s.l., which accounted for 5% of the specimens collected in CDC LTs and 11% of those collected during HLCs. Monthly collection totals, grouped by village IRS status, for these species groups are presented in Tables 2, 3 and Fig. 3 (note that each study arm had an equal number of collection nights each month). Additional morphological identifications included *Anopheles tenebrosus* (n = 168), *Anopheles coustani* (n = 135), *Anopheles ziemanni* (n = 100), *Anopheles squamosus/cydippis* (n = 25), *Anopheles pretoriensis* (n = 11), *Anopheles rufipes* (n = 8), *Anopheles pharoensis* (n = 7), *Anopheles caliginosus* (n = 4), *Anopheles natalensis* (n = 4), other *Anopheles* spp*.* (n = 3) (Additional file 1: Table S1).

**Table 2:** Total number of *Anopheles funestus* s.l. and *Anopheles gambiae* s.l. collected in indoor CDC LTs during the cluster-randomized trial, by month and village IRS status

Month	CDC LT			
	*An. funestus* s.l		*An. gambiae* s.l	
	No-IRS	IRS	No-IRS	IRS
Sep-16	24	346	3	2
Oct-16	112	156	1	14
*Nov-16*	*32*	*3*	*1*	*0*
*Dec-16*	*106*	*109*	*21*	*55*
Jan-17	–	–	–	–
Feb-17	–	–	–	–
Mar-17	1013	88	77	70
Apr-17	3915	1579	182	87
May-17	2734	1148	18	19
Jun-17	1150	456	4	1
Jul-17	664	253	2	0
Aug-17	562	287	1	2
Sep-17	394	244	1	0
Oct-17	125	291	0	3
*Nov-17*	*196*	*71*	*1*	*1*
*Dec-17*	*428*	*131*	*8*	*2*
Jan-18	536	56	201	36
Feb-18	441	104	26	4
Mar-18	752	53	86	22
Apr-18	–	–	–	–
May-18	903	108	16	2
Jun-18	710	112	6	4
Jul-18	302	64	4	3
Aug-18	174	37	0	4
Sep-18	207	51	0	1
Oct-18	245	90	36	22

Months highlighted in italics show the duration of the IRS campaigns

*CDC LT* US Centers for Disease Control light trap, *IRS* indoor residual spraying

**Table 3:** Total number of *Anopheles funestus* s.l. and *Anopheles gambiae* s.l. collected by HLC during the cluster-randomized trial, by month, IRS status, and collector position

Month	HLC						HLC					
	*An. funestus* s.l						*An. gambiae* s.l					
	No-IRS			IRS			No-IRS			IRS		
	Total	Indoor	Outdoor	Total	Indoor	Outdoor	Total	Indoor	Outdoor	Total	Indoor	Outdoor
Sep-16	18	9	9	57	33	24	2	0	2	1	1	0
Oct-16	2	1	1	5	2	3	2	2	0	1	0	1
*Nov-16*	*4*	*3*	*1*	*5*	*3*	*2*	*0*	*0*	*0*	*2*	*0*	*2*
*Dec-16*	*3*	*0*	*3*	*8*	*6*	*2*	*0*	*0*	*0*	*16*	*6*	*10*
Jan-17	–	–	–	–	–	–	–	–	–	–	–	–
Feb-17	–	–	–	–	–	–	–	–	–	–	–	–
Mar-17	51	22	29	2	1	1	12	4	8	7	7	0
Apr-17	244	119	125	31	18	13	25	18	7	2	1	1
May-17	601	266	335	51	19	32	3	1	2	0	0	0
Jun-17	273	145	128	0	0	0	0	0	0	0	0	0
Jul-17	310	178	132	10	4	6	0	0	0	0	0	0
Aug-17	56	14	42	14	2	12	0	0	0	0	0	0
Sep-17	64	28	36	8	6	2	0	0	0	0	0	0
Oct-17	15	5	10	0	0	0	1	0	1	0	0	0
*Nov-17*	*6*	*3*	*3*	*0*	*0*	*0*	*0*	*0*	*0*	*0*	*0*	*0*
*Dec-17*	*18*	*6*	*12*	*7*	*4*	*3*	*0*	*0*	*0*	*0*	*0*	*0*
Jan-18	63	42	21	10	4	6	57	12	45	27	6	21
Feb-18	24	11	13	36	23	13	27	12	15	13	4	9
Mar-18	49	40	9	12	7	5	56	13	43	10	3	7
Apr-18	–	–	–	–	–	–	–	–	–	–	–	–
May-18	73	54	19	6	4	2	0	0	0	0	0	0
Jun-18	85	38	47	7	7	0	4	3	1	0	0	0
Jul-18	21	16	5	11	5	6	0	0	0	0	0	0
Aug-18	24	11	13	8	4	4	2	1	1	0	0	0
Sep-18	73	36	37	4	3	1	0	0	0	0	0	0
Oct-18	34	14	20	9	0	9	0	0	0	1	0	1

See text for an explanation of the missing data points. Months highlighted in italics show the duration of the IRS campaigns

*HLC* human landing collection, *IRS* indoor residual spraying

A subsample of 6216 *Anopheles* mosquitoes were screened via species-diagnostic PCR and/or DNA sequencing, including 5197 specimens morphologically identified as *An. funestus* s.l., 798 morphologically identified as *An. gambiae* s.l., 79 morphologically identified as *An. coustani* s.l., and 142 variously identified as other *Anopheles* species (Additional file 1: Table S2). Of this subsample, 82% of the specimens (n = 5070) were able to be identified to species while the remaining 18% (n = 1146) gave poor results, mostly due to fungal contamination. Of the *An. funestus* group specimens that were able to be identified to species, 96.5% (n = 4099) were *An. funestus *sensu stricto (s.s.), with the remaining specimens comprising *Anopheles rivulorum* (n = 119), *Anopheles vandeeni* (n = 24), *Anopheles parensis* (n = 2), *An. rivulorum*-like (n = 1), and one *An. funestus* × *An. rivulorum* hybrid (as evidenced by conflicting mitochondrial and nuclear specimen signatures). Of the *An. gambiae* complex specimens able to be identified to species, 81.3% were *Anopheles arabiensis* (n = 529), 13.1% were *An. gambiae* s.s. (n = 85), and 5.1% were *Anopheles coluzzii* (n = 33).

The head-thorax portions of the mosquitoes subsampled for PCR analysis were also screened for *P. falciparum* and *P. vivax* DNA. In total, 146 (2.4%) tested positive for *P. falciparum* and 24 (0.4%) were positive for *P. vivax*; three of these specimens were coinfected with both malaria parasite species. Of the 167 *Plasmodium*-positive mosquitoes, 162 (97.0%) belonged to the *An. funestus* group, three (1.8%) to the *An. gambiae* complex, and two (1.2%) to the *An. coustani* group (Additional file 1: Table S2).

For those *P. falciparum*-positive mosquitoes that were further identifiable to species level via PCR (total n = 131), 94.7% (n = 124) were *An. funestus* s.s., 2.3% were *An. rivulorum* (n = 3), 1.5% (n = 2) were *An. gambiae* s.s., 0.8% (n = 1) were *An. coustani* s.s., and 0.8% (n = 1) were *Anopheles namibiensis*. The vector species composition was similar when considering the *P. vivax*-positive specimens (total n = 21): 75% (n = 18) were *An. funestus* s.s., 4.2% (n = 1) were *An. rivulorum*, and 8.3% (n = 2) were *An. gambiae* s.s. Of the three specimens that were coinfected with both *Plasmodium* species, two were *An. funestus* s.s. and one was *An. gambiae* s.s. (Additional file 1: Table S2).

Results of the WHO tube tests indicate that *An. funestus* s.l. from Mopeia were resistant to pyrethroids, and that the frequency of resistant phenotypes in this population likely increased during the trial [26, 27]: mortality rates of 86% with alpha-cypermethrin and 88% with deltamethrin were recorded in October 2017, the midpoint of the study, but by the end of the study in September 2018 mortality with alpha-cypermethrin had fallen to 7% (deltamethrin was not tested in 2018) (Table 4). By contrast, complete susceptibility (100% mortality) was observed against PM in both years (Table 4). For reference, though not a primary vector of malaria during the trial, *An. gambiae* s.l. from Mopeia also showed 100% mortality against PM in both years and emerging resistance to alpha-cypermethrin (100% mortality in 2017; 91% mortality in 2018) (Table 4).

**Table 4:** Insecticide susceptibility in the target vector populations during the cluster-randomized trial. Adapted from the PMI VectorLink Project Mozambique Entomological Monitoring Reports [26, 27]

*Anopheles* species	Year	Insecticides	24 h % mortality (no. tested)	Status
*An. funestus* s.l	2017	Alpha-cypermethrin	86 (100)	Resistance
		Bendiocarb	89 (100)	Resistance
		DDT	100 (100)	Susceptible
		Deltamethrin	88 (100)	Resistance
		Pirimiphos-methyl	100 (100)	Susceptible
	2018	Alpha-cypermethrin	7 (100)	Resistance
		Pirimiphos-methyl	100 (100)	Susceptible
*An. gambiae* s.l	2017	Alpha-cypermethrin	100 (100)	Susceptible
		Pirimiphos-methyl	100 (100)	Susceptible
	2018	Alpha-cypermethrin	91 (100)	Possible resistance
		Pirimiphos-methyl	100 (100)	Susceptible

*DDT* dichloro-diphenyl-trichloroethane, *PBO* piperonyl butoxide

### Impact of the intervention on vector densities—indoor CDC LT collections

**Fig. 4:**
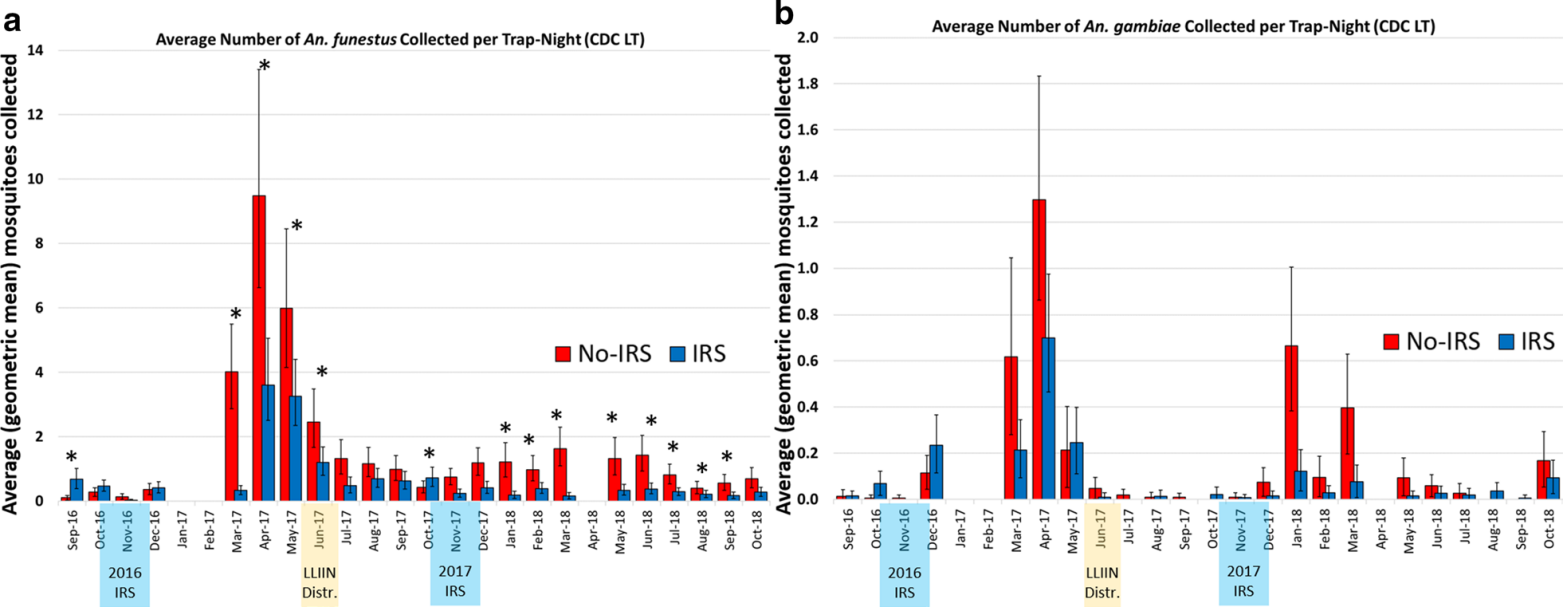
Monthly trends in the average number of mosquitoes collected per trap-night in indoor human-baited CDC LTs during the cluster-randomized trial for **a** *An. funestus* s.l. and **b** *An. gambiae* s.l. Geometric means are presented. In **a**, * indicates mosquito collection IRRs that are significantly different from 1 (zero-inflated negative binomial regression model, *p* < 0.05). The number of *An. gambiae* s.l. collected was too low to enable a similarly meaningful statistical analysis, but the trends are similar. Note the different scales on the y-axes that reflect much higher densities (almost ×20) of *An. funestus* s.l. than *An. gambiae* s.l. *CDC LT* US Centers for Disease Control light trap, *IRR* incidence rate ratio, *IRS* indoor residual spraying

Monthly trends in the average number of mosquitoes collected per trap-night for *An. funestus* s.l. and *An. gambiae* s.l. are presented in Tables 5, 6 and Fig. 4. Monthly averages are visualized using the geometric mean number of mosquitoes collected per trap-night with 95% confidence intervals (Fig. 4). Tables 5, 6 also shows formal comparisons of mosquito densities across IRS and no-IRS study arms using incidence rate ratios with associated *p*-values that describe the probability that the true IRR is equal to 1 (which would indicate no difference in mosquito density across study arms).

**Table 5:** The average number of *Anopheles funestus* s.l. collected per CDC LT collection-night during the cluster-randomized trial, by IRS status

Month	Average number of mosquitoes collected per trap-night (95% CI)		IRR^a^	p^b^
	No-IRS	IRS		
Sep-16	0.10 (0.03–0.18)	0.68 (0.39–1.02)	4.12	< 0.001
Oct-16	0.28 (0.15–0.42)	0.47 (0.30–0.65)	0.90	0.832
*Nov-16*	*0.14 (0.06–0.22)*	*0.02 (0.00–0.05)*	*0.41*	*0.299*
*Dec-16*	*0.37 (0.20–0.55)*	*0.41 (0.25–0.60)*	*0.82*	*0.542*
Jan-17	–	–	–	–
Feb-17	–	–	–	–
Mar-17	4.01 (2.87–5.50)	0.34 (0.21–0.48)	0.21	< 0.001
Apr-17	9.47 (6.62–13.40)	3.61 (2.51–5.06)	0.45	0.006
May-17	5.98 (4.15–8.45)	3.25 (2.35–4.40)	0.44	0.004
Jun-17	2.46 (1.67–3.49)	1.20 (0.80–1.69)	0.53	0.004
Jul-17	1.32 (0.85–1.91)	0.48 (0.26–0.75)	0.75	0.364
Aug-17	1.17 (0.76–1.67)	0.70 (0.43–1.01)	0.70	0.270
Sep-17	0.99 (0.64–1.41)	0.63 (0.38–0.92)	0.85	0.553
Oct-17	0.43 (0.26–0.62)	0.73 (0.45–1.06)	1.82	0.016
*Nov-17*	0.74 (0.51–1.02)	0.24 (0.12–0.37)	0.88	0.635
*Dec-17*	*1.19 (0.80–1.66)*	*0.41 (0.24–0.61)*	*0.58*	*0.095*
Jan-18	*1.22 (0.75–1.81)*	*0.18 (0.08–0.30)*	*0.29*	*< 0.001*
Feb-18	0.98 (0.63–1.41)	0.39 (0.23–0.57)	0.36	< 0.001
Mar-18	1.63 (1.09–2.30)	0.16 (0.06–0.27)	0.30	< 0.001
Apr-18	–	–	–	–
May-18	1.32 (0.81–1.97)	0.34 (0.18–0.52)	0.22	< 0.001
Jun-18	1.42 (0.93–2.04)	0.38 (0.22–0.56)	0.32	0.001
Jul-18	0.81 (0.53–1.15)	0.29 (0.17–0.41)	0.35	0.001
Aug-18	0.40 (0.22–0.61)	0.22 (0.11–0.34)	0.33	0.017
Sep-18	0.56 (0.34–0.83)	0.18 (0.08–0.28)	0.48	0.001
Oct-18	0.70 (0.41–1.04)	0.29 (0.15–0.43)	0.52	0.123
Overall^c^	1.34 (1.22–2.46)	0.57 (0.52–0.62)	0.52	< 0.001

The geometric mean number (with 95% CI) of mosquitoes by month are presented. Months highlighted in italics show the duration of the IRS campaigns

*CDC LT* US Centers for Disease Control light trap, *IRR* incidence rate ratio, *IRS* indoor residual spraying

^a^Incidence rate ratio from the zero-inflated negative binomial regression analysis, describing the rate at which mosquitoes were collected in IRS villages relative to no-IRS villages. An IRR = 1 indicates no difference across study arms

^b^The probability that the true IRR = 1; zero-inflated negative binomial regression

^c^Overall averages from the duration of the study after the first IRS campaign, December 2016–October 2018

**Table 6:** The average number of *Anopheles gambiae* s.l. collected per CDC LT collection-night during the cluster-randomized trial, by village IRS status

Month	Average number of mosquitoes collected per trap-night (95% CI)		IRR^a^	p^b^
	No-IRS	IRS		
Sep-16	0.01 (0.00–0.04)	0.02 (0.00–0.04)	0.33	< 0.001
Oct-16	0.01 (0.00–0.02)	0.07 (0.02–0.12)	2.00	< 0.001
*Nov-16*	*0.01 (0.00–0.02)*	*0.00 (0.00–0.00)*	*1.00*	*NR*
*Dec-16*	*0.11 (0.04–0.19)*	*0.23 (0.11–0.37)*	*1.60*	*0.247*
Jan-17	–	–	–	–
Feb-17	–	–	–	–
Mar-17	0.62 (0.28–1.05)	0.21 (0.09–0.35)	0.97	0.953
Apr-17	1.30 (0.86–1.83)	0.70 (0.46–0.97)	0.53	0.083
May-17	0.21 (0.05–0.40)	0.25 (0.11–0.40)	0.65	0.241
Jun-17	0.05 (0.00–0.10)	0.01 (0.00–0.03)	1.00	NR
Jul-17	0.02 (0.00–0.04)	0.00 (0.00–0.00)	1.00	NR
Aug-17	0.01 (0.00–0.03)	0.01 (0.00–0.04)	2.00	< 0.001
Sep-17	0.01 (0.00–0.03)	0.00 (0.00–0.00)	1.00	NR
Oct-17	0.00 (0.00–0.00)	0.02 (0.00–0.05)	1.00	NR
*Nov-17*	*0.01 (0.00–0.03)*	*0.01 (0.00–0.02)*	*1.00*	*NR*
*Dec-17*	*0.07 (0.01–0.14)*	*0.01 (0.00–0.04)*	*0.75*	*0.048*
Jan-18	0.67 (0.38–1.00)	0.12 (0.04–0.22)	0.52	0.015
Feb-18	0.10 (0.01–0.19)	0.03 (0.00–0.06)	0.23	< 0.001
Mar-18	0.40 (0.20–0.63)	0.08 (0.01–0.15)	0.97	0.931
Apr-18	–	–	–	–
May-18	0.09 (0.02–0.18)	0.02 (0.00–0.04)	0.37	< 0.001
Jun-18	0.06 (0.01–0.11)	0.03 (0.00–0.06)	1.33	0.162
Jul-18	0.03 (0.00–0.07)	0.02 (0.00–0.05)	0.75	0.501
Aug-18	0.00 (0.00–0.00)	0.04 (0.00–0.07)	1.00	NR
Sep-18	0.00 (0.00–0.00)	0.01 (0.00–0.02)	1.00	NR
Oct-18	0.17 (0.05–0.29)	0.09 (0.02–0.17)	0.76	0.499
Overall^c^	0.15 (0.13–0.18)	0.08 (0.07–0.10)	0.66	0.019

The geometric mean number (with 95% CI) of mosquitoes by month are presented. Months highlighted in italics show the duration of the IRS campaigns

*CDC LT* US Centers for Disease Control light trap, *IRR* incidence rate ratio, *IRS* indoor residual spraying

Incidence ratio from the zero-inflated negative binomial regression analysis, describing the rate at which mosquitoes were collected in IRS villages relative to no-IRS villages. An IRR = 1 indicates no difference across study arms

^b^The probability that the true IRR = 1; zero-inflated negative binomial regression

^c^Overall averages from the duration of the study after the first IRS campaign, December 2016–October 2018

Baseline data from September and October 2016 suggest that, prior to the initial IRS campaign, mosquito densities of both *An. funestus* s.l. and *An. gambiae* s.l. were significantly higher in the IRS villages. This trend reversed dramatically upon IRS implementation, after which the overall IRR for *An. funestus* s.l. was 0.52 (CI_95_ 0.41–0.67; *p* < 0.001)—indicating a significant reduction of 48% (CI_95_ 33–59%) in the average number of *An. funestus* s.l. collected per night over the duration of the study (Table 5). For *An. gambiae* s.l., this IRR was 0.66 (CI_95_ 0.47–0.93; *p* < 0.019), indicating a significant overall reduction of around 33% (CI_95_ 7–53%) over the duration of the study (Table 6).

The impact of the IRS campaigns was greatest in the months immediately after the conclusion of spray operations, waning over time (Fig. 4a and Table 5). After the first IRS campaign in 2016, the reduction in *An. funestus* s.l. densities observed in the IRS clusters remained statistically significant for 6 months, through June 2017, while in year 2 the reductions remained significant for 9 months, until September 2018. Considering the months of highest mosquito density and greatest intervention impact (which also correspond to peak transmission) suggests a peak reduction of an average of 60% fewer mosquitoes (CI_95_ 33–75%) collected per trap-night after the 2017 campaign (March–June) and, on average, 70% fewer mosquitoes collected (CI_95_ 47–83%) per trap-night after the 2018 campaign (January–September).

Relatively few *An. gambiae* s.l. were collected throughout the study, and the number of collection-nights with zero mosquitoes introduces substantial variability in the monthly estimates (Table 6; Fig. 4b). Though the overall trend of IRS dramatically reducing mosquito densities is consistent for this species, statistical results should be interpreted with caution as the sampling strategy was not likely powered sufficiently to allow robust comparisons of monthly trends in *An. gambiae* s.l. densities.

### Impact of the intervention on vector densities and feeding behaviours—HLCs

**Fig. 5:**
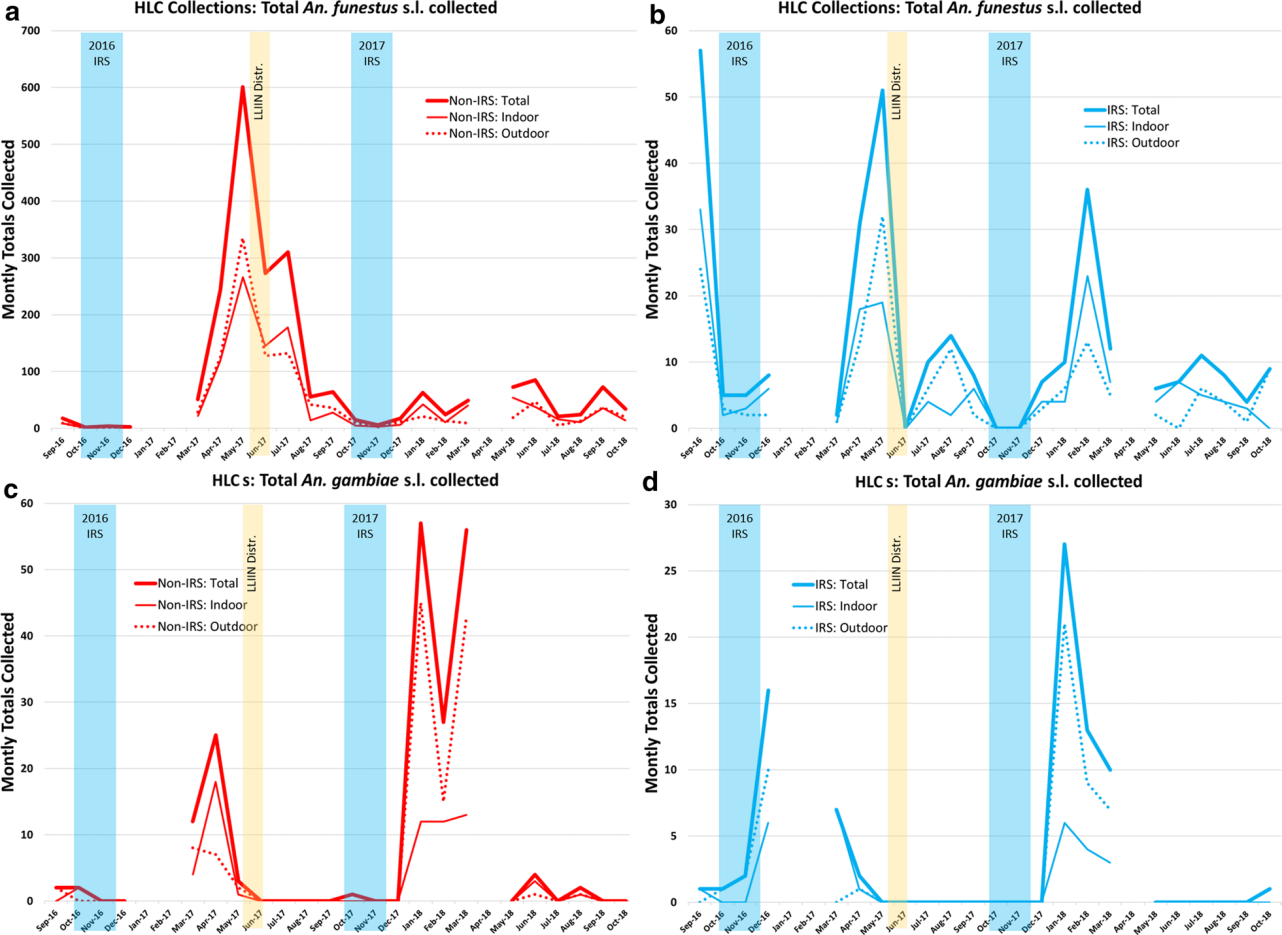
The total number of mosquitoes collected during paired HLCs during the cluster-randomized trial, by collector position, and by IRS status. **a** *Anopheles funestus* collected in no-IRS clusters; **b** *An. funestus* collected in IRS clusters. **c** *Anopheles gambiae* collected in no-IRS clusters; **d** *An. gambiae* collected in IRS clusters. *HLC* human landing collection, *IRS* indoor residual spraying

**Fig. 6:**
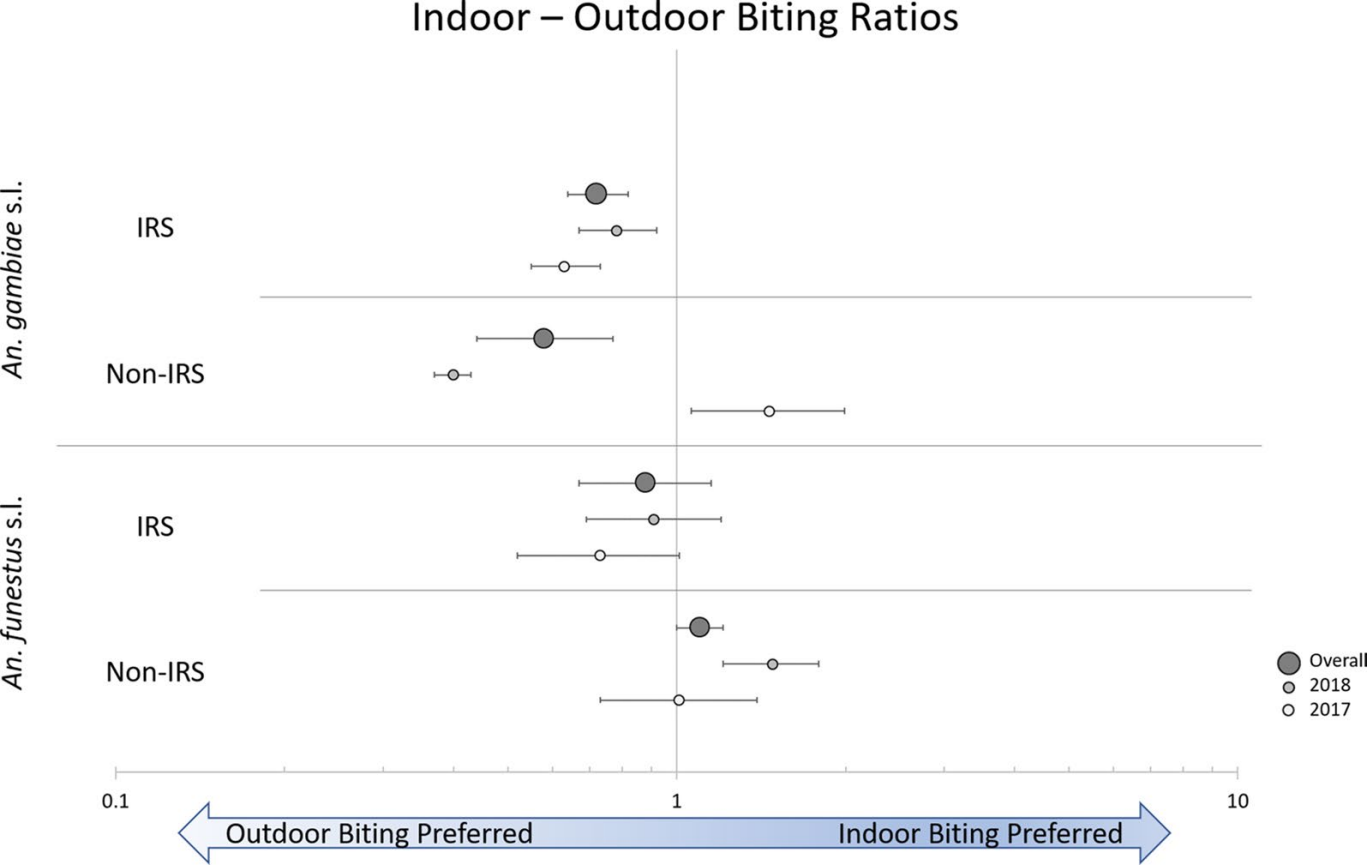
Indoor-outdoor biting ratios observed during HLCs throughout the cluster-randomized trial. Shown is the ratio of mosquitoes collected indoors to outdoors. *HLC* human landing collection, *IRS* indoor residual spraying

The total number of *An. funestus* s.l. and *An. gambiae* s.l. collected by HLC (Table 3) are presented by collector position (indoor and outdoor) in Fig. 5. For *An. funestus* s.l., the overall indoor to outdoor biting ratio for the duration of the study was 1.10 (CI_95_ 1.00–1.21; *p* = 0.045) in no-IRS villages and 0.88 (CI_95_ 0.67–1.15; *p* = 0.388) in IRS villages (Fig. 6). This indicates a slight preference for indoor feeding in the absence of IRS, though the trend was somewhat variable by year and by spray status (Fig. 6) and outdoor feeding was almost as likely throughout both study arms. Feeding behaviours in *An. gambiae* s.l. were also somewhat variable, but an overall tendency to prefer biting outdoors was observed in both no-IRS villages (ratio = 0.58 [CI_95_ 0.44–0.77; *p* < 0.001]) and IRS villages (ratio = 0.73 [CI_95_ 0.65–0.83; *p* < 0.001]) (Fig. 6).

**Fig. 7:**
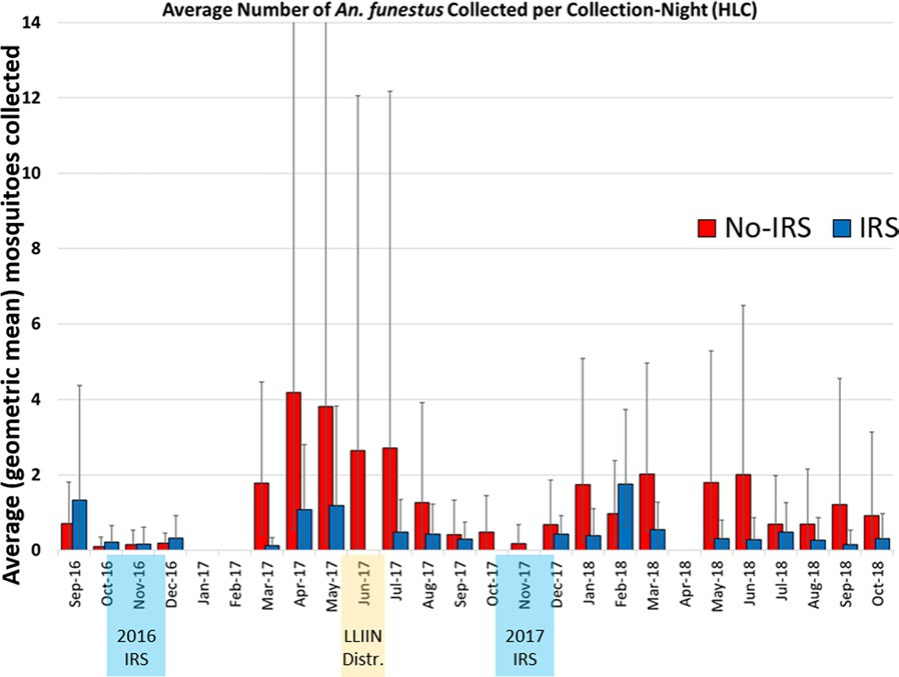
Monthly trends in the average nightly number of mosquitoes collected during HLCs for *An. funestus* s.l. Geometric means of total mosquitoes collected (indoors + outdoors) by collection-night are presented. *HLC* human landing collection, *IRS* indoor residual spraying

The number of *An. funestus* s.l. collected during HLC surveillance was significantly lower in IRS villages compared to no-IRS villages, and the impact of the intervention was consistent both indoors and outdoors: the IRR was 0.25 (CI_95_ 0.12–0.53; *p* < 0.001) indoors and 0.32 (CI_95_ 0.13–0.78; *p* = 0.012) outdoors. Accordingly, the monthly trends in average number of mosquitoes collected per HLC collection-night are presented as the aggregate totals from indoor and outdoor positions at the same house. These trends are presented in Table 7 and Fig. 7. After IRS implementation, the overall IRR for *An. funestus* s.l. was 0.26 (CI_95_ 0.10–0.62; *p* = 0.010)—indicating a significant reduction of 74% (CI_95_ 38–90%) in the average number of mosquitoes collected per night over the duration of the study (Table 7; Fig. 7).

**Table 7:** The average number of *Anopheles funestus* collected per collection-night (HLC)

Month	Average number of mosquitoes collected per collection-night (95% CI)		IRR^a^	p^b^
	No-IRS	IRS		
Sep-16	0.70 (0.03–1.81)	1.33 (0.01–4.37)	3.96	0.021
Oct-16	0.10 (0.00–0.34)	0.21 (0.00–0.65)	1.25	< 0.001
*Nov-16*	*0.14 (0.00–0.54)*	*0.16 (0.00–0.61)*	*1.25*	*< 0.001*
*Dec-16*	*0.19 (0.00–0.45)*	*0.32 (0.00–0.92)*	*2.67*	*< 0.001*
Jan-17	–	–	–	–
Feb-17	–	–	–	–
Mar-17	1.79 (0.42–4.47)	0.12 (0.00–0.33)	0.16	0.002
Apr-17	4.19 (0.55–16.35)	1.08 (0.13–2.80)	0.17	0.046
May-17	3.81 (0.16–19.03)	1.18 (0.00–3.82)	0.15	0.018
Jun-17	2.65 (0.02–12.06)	0.00 (0.00–0.00)	NR	NR
Jul-17	2.71 (0.05–12.18)	0.48 (0.00–1.35)	0.05	< 0.001
Aug-17	1.26 (0.04–3.92)	0.42 (0.00–1.22)	0.33	< 0.001
Sep-17	0.41 (0.00–1.33)	0.29 (0.00–0.74)	0.08	< 0.001
Oct-17	0.48 (0.00–1.45)	0.00 (0.00–0.00)	NR	NR
*Nov-17*	*0.18 (0.00–0.68)*	*0.00 (0.00–0.00)*	*NR*	*NR*
*Dec-17*	*0.68 (0.00–1.87)*	*0.43 (0.07–0.91)*	*0.31*	*< 0.001*
Jan-18	1.74 (0.23–5.09)	0.38 (0.00–1.10)	0.32	0.007
Feb-18	0.97 (0.15–2.38)	1.76 (0.61–3.74)	1.17	0.824
Mar-18	2.01 (0.52–4.97)	0.55 (0.06–1.28)	0.39	0.071
Apr-18	–	–	–	–
May-18	1.79 (0.24–5.29)	0.30 (0.00–0.79)	0.19	0.022
Jun-18	2.01 (0.21–6.49)	0.28 (0.00–0.86)	0.25	0.007
Jul-18	0.69 (0.00–1.97)	0.48 (0.00–1.26)	0.52	0.080
Aug-18	0.69 (0.00–2.16)	0.26 (0.00–0.86)	0.50	< 0.001
Sep-18	1.22 (0.00–4.56)	0.14 (0.00–0.54)	0.16	< 0.001
Oct-18	0.91 (0.00–3.13)	0.30 (0.00–0.97)	0.40	< 0.001
Overall^c^	1.10 (0.82–1.43)	0.43 (0.31–0.56)	0.26	0.010

The geometric mean number (with 95% CI) of mosquitoes by month are presented. Months highlighted in italics show the duration of the IRS campaigns

*HLC* human landing collection, *IRR* incidence rate ratio, *IRS* indoor residual spraying

^a^The incidence ratio describing the rate at which mosquitoes were collected in IRS villages relative to no-IRS villages. An IRR = 1 indicates no difference across study arms

^b^The probability that the true IRR = 1; zero-inflated negative binomial regression

^c^Overall averages from the duration of the study after the first IRS campaign, December 2016–October 2018

As with the CDC LT collections, trends in HLC results also suggest substantially reduced densities of *An. gambiae* s.l. following IRS, though so few total *An. gambiae* s.l. were collected (542 total over 26 months of surveillance) the study was not sufficiently powered to detect differences across study arms for this species and the reductions observed were not statistically significant in any month.

### Entomological inoculation rate

Though more than 6000 mosquito specimens were screened for *Plasmodium* DNA, the uneven degradation of specimens during storage and shipment meant that the vast majority of these samples came from indoor CDC LT collections and were not well distributed by month or by intervention status: for example, sporozoite rate estimates are only available for 14 months (March 2017 through May 2018) and more than half (52%) of all *P. falciparum*-positive mosquitoes were collected from a single village on three consecutive nights in April 2017. Accordingly, estimates of sporozoite rates (and resultant entomological inoculation rates) vary substantially over time and by intervention status and thus remain difficult to interpret. Nonetheless, crude estimates of monthly entomological inoculation rates (EIRs) were calculated by multiplying monthly *An. funestus* s.l. sporozoite rates by the corresponding average number of *An. funestus* s.l. collected per CDC LT collection-night, per village (Additional file 1: Table S3). While not the preferred method for estimating EIR [38], this approach takes advantage of the fact that surveillance results from the indoor, human-baited CDC LT collections were much more robust than the results of the HLCs, both in terms of overall number of mosquitoes collected and the overall number of collection points (Table 7; Fig. 4). It was also assumed that all of *An. funestus* s.l. collected were actively seeking a blood meal and would have bitten once on the night in which they were trapped. As such, the estimated average (geometric mean) number of *P. falciparum* infectious bites in no-IRS villages ranged from 0.00 per household per month in November 2017 to 2.82 per household per month in October 2017 and in IRS villages from 0.00 per household per month in multiple months to 5.33 per household per month in April 2017 (Additional file 1: Table S3, Fig. S1). Although the confidence intervals associated with these monthly estimates are large (Additional file 1: Table S3, Fig. S1), the overall average EIR estimate for IRS villages was 0.28 (CI_95_ 0.08–0.60) infectious bites per household per month and for no-IRS villages was 0.57 (CI_95_ 0.28–1.00) infectious bites per household per month, suggesting a trend of nearly 50% lower EIR in IRS *versus* no-IRS villages, though the difference is not statistically significant (Additional file 1: Fig. S1).

## Discussion

Collectively, these results provide compelling evidence that during the study (1) mosquitoes from the *An. funestus* group (almost exclusively *An. funestus* s.s.) were the dominant vector of both *P. falciparum* and *P. vivax*, (2) this population of *An. funestus* demonstrated variable and likely increasing resistance to pyrethroids, (3) *An. funestus* s.l. vectors were equally likely to bite indoors or outdoors, (4) the overall effect of IRS on decreasing *An. funestus* s.l. densities and biting rates was substantial and consistent in both years, and (5) the impact of IRS on reducing observed biting rates was consistent indoors and outdoors.

Some of the limitations of the entomological surveillance component of this cluster-randomized trial include the relatively small number of surveillance sites (ten CDC LT collection sites per treatment arm), the relatively low number of mosquitoes that were collected during HLC activities, and the lack of temporal and geographic diversity represented in the samples that were available for molecular screening. Indeed, the lack of statistical significance in the effect estimate for reduced EIR is most likely because of these limitations and the challenges they present in consistently estimating sporozoite rates throughout the study, and should not be interpreted as evidence of a weak effect of IRS on malaria transmission. Additionally, the gaps in surveillance data included several months of expected peak impact (January–February 2017, April 2018), which may cause aggregate reductions in mosquito density to be underestimated.

Another limitation of the present work involves challenges in interpreting the insecticide susceptibility test results for the primary vector species, *An. funestus*. While the preferred method of testing local mosquito populations for insecticide resistance involves using adult mosquitoes of a standardized age and physiological status recently emerged from wild-caught larvae reared to adulthood in a field laboratory, the scarcity of *An. funestus* larvae encountered during these surveillance activities required the use of relatively few wild-caught adult specimens collected via Prokopack aspiration. This resulted in a limited number of test mosquitoes, of variable age and physiological status and pooled from various collection points, available for a limited number of bioassays. While pyrethroid resistance in *An. funestus* is clearly present in Mopeia, and very likely to have increased in frequency during the trial, its prevalence, intensity, and fine-scale temporal and geospatial variability over the duration of the 2-year study remain difficult to assess.

The molecular screening results suggest an overall malaria transmission landscape in Mopeia that is rich in species diversity, both in terms of vectors and parasites. However, *An. funestus* s.s. accounted for nearly 95% of all *Plasmodium*-positive specimens and more than 90% of all adult *Anopheles* collected during the study, firmly implicating *An. funestus* s.s. as the dominant vector species during the trial. Interestingly, trends in reduced density and biting rates observed in the local *An. gambiae* s.l. population (which was predominantly *An. arabiensis*) were similar to those observed in the local *An. funestus* s.l. population, suggesting a similar impact of IRS on these mosquitoes even though this population was more likely to prefer feeding outdoors. While this is encouraging, it is important to emphasize that the overall number of *An. gambiae* s.l. collected during the study was low (less than 5% of the total *Anopheles* collected), preventing any robust statistical analysis of these trends. Finally, while there is no evidence that *An. arabiensis* was contributing to malaria transmission in Mopeia during the trial, *An. gambiae* s.s., *An. rivulorum*, and *An. coustani* s.s., were probable secondary vectors. Surveillance activities should continue to longitudinally monitor these populations closely to assess their contribution to local transmission and how they may respond to various malaria control interventions over time. It is also interesting to note that one *P. falciparum* PCR-positive specimen, originally identified morphologically as *An. tenebrosus*, was subsequently identified as *An. namibiensis* based ITS2 gene sequencing results. Similarly, *An. vandeeni*, *An. rivulorum*-like, and *An*. *coluzzii* have not been widely reported previously in Mozambique—the presence of these species, and any possible role as secondary malaria vectors, should be confirmed and studied further.

The overall impact estimates, which are aggregate estimates that consider the duration of the study period, indicate *An*. *funestus* s.l. catch reductions of around 50% in CDC LTs (Table 7) and of around 75% during HLCs. This effect was consistent in both years, and was greatest in the months that closely followed IRS operations and waned over time. Considering that these months of high entomological impact also correspond with the months of high mosquito density, it is possible that these aggregate estimates underestimate the true impact of the IRS intervention during the trial. Indeed, the effect reached as high as an 80% reduction in indoor densities (Table 5) and a 90% reduction in biting estimates (Table 7) during some of the months of peak transmission potential.

It is also encouraging that the impact of IRS on reducing exposure to malaria vectors remained statistically significant for a minimum of 6 months (in 2017) and as long as 9 months (in 2018)—even though standard interpretation of the WHO cone wall bioassay results (in which the effectiveness of a residual insecticide on a wall is indicated by 24-h mortality greater than 80% in a test strain of laboratory-reared mosquitoes after a 30-min wall exposure) suggested waning efficacy after just 3 months. Though the wall bioassay is a critically important tool for evaluating spray campaign quality and comparing residual effects of IRS across different scenarios, exactly how the standard interpretation of wall bioassay results relate to operationally effective vector control is not clear. Results from this trial suggest that the true residual effectiveness of some IRS products may be routinely underestimated when determined exclusively using the WHO cone wall bioassays. In areas of high coverage, IRS seems to continue providing community protection even after mosquito test mortality drops to less than the 80% threshold as measured by cone bioassays, a key observation that should be further validated in various transmission settings.

Variability was noted in the indoor-outdoor biting ratios observed in *An. funestus* s.l. during HLC activities. In no-IRS villages, the indoor-outdoor biting ratio was 1.01 (CI_95_ 0.73–1.39; *p* = 0.956) in the first year and 1.48 (CI_95_ 1.21–1.79; *p* < 0.001) in the second year, indicating a preference for indoor feeding that is evident in January, March, and May of 2018 but relatively inconsistent otherwise. In IRS clusters, neither year’s ratio was significantly different from 1.0, though a tendency to be slightly more exophagic in IRS clusters compared to no-IRS clusters is suggested (Fig. 6). Continued monitoring of subsequent IRS campaigns could help explore if and how these trends are changing. In addition, given the comparatively low density of mosquitoes collected during HLC activities relative to CDC LT collections, it is not surprising that very few of the *P. falciparum*-positive mosquitoes were collected during HLC activities. This complicates any robust analysis of differences in EIR that may have existed indoors versus outdoors during the study. That said, it is interesting to note that of the 18 total *P. falciparum*-positive mosquitoes that originated from HLCs, 12 were collected outdoors and six were collected indoors, suggesting that transmission in Mopeia was occurring both indoors and outdoors. It is likely that a single population of *An. funestus* s.s. was feeding opportunistically in both locations and was the primary driver of malaria transmission during the study. Considering this, the observation that IRS reduced *An. funestus* s.l. biting rates both indoors and outdoors is encouraging, but whether or not these outdoor biting behaviours may eventually become refractory to otherwise effective IRS and/or LLIN interventions, and thereby the degree to which they could contribute to residual transmission, is unclear and should be further assessed.

In both IRS and no-IRS sites, overall mosquito densities for *An. funestus* s.l. and *An. gambiae* s.l. were lower (by 50%) in 2018 than in 2017. It is possible that this was the result of some combination of natural seasonal variation and the general impact of the 2017 LLIN distribution campaign. It is also apparent that the reduction in vector densities observable after IRS remained statistically significant for a longer period of time in 2018 (9 months) compared to 2017 (6 months). It is possible that there was an additive effect when implementing the repeated, annual IRS campaigns, at least in terms of extending the impact for some additional months—though because overall peak mosquito densities were much lower in 2018 compared to 2017, it could be that the impact of the IRS intervention is simply more apparent under these lower-density conditions.

Similarly, it is compelling that the effects of the IRS campaigns on reducing *An. funestus* s.l. densities and biting rates were consistent in each year—including study year 2, when standard LLIN ownership and use estimates were both above 90% and WHO tube bioassays indicated low to moderate resistance with an initial mortality of 86% against alpha-cypermethrin. Given these conditions, at least during the initial months of the second transmission season, the new LLINs used in Mopeia would have been much closer to maximum insecticidal effectiveness than at the end of the trial, when mortality was only 7% in the same bioassays. That the impact of IRS is clearly evident regardless suggests little redundancy when implementing the two core vector control interventions together, even while the LLINs were expected to be effective at killing mosquitoes on their own. That said, it is important to acknowledge that this study was not designed to estimate the effectiveness of the LLINs alone and it is also highly likely that some of the additional impact observed when combining interventions resulted from IRS with an effective chemical compensating for some loss of LLIN effectiveness in the light of expanding pyrethroid resistance. While the ultimate public health impact of any vector control intervention is extraordinarily complex and dependent on multiple factors, it is encouraging that the impact of the IRS campaigns conducted during this study were equally apparent in both years, regardless of baseline mosquito population densities, a concurrent mass LLIN distribution campaign, and a shifting pyrethroid resistance profile.

## Conclusion

Collectively, these entomological surveillance results show that the 3GIRS campaigns of 2016 and 2017 had a substantial impact on reducing human exposure to malaria vectors in Mopeia—a high-burden district in central Mozambique, where the primary vector, *An. funestus* s.s., is equally likely to feed indoors or outdoors and demonstrated moderate to high pyrethroid resistance. Plans for future analyses include exploration of how these entomological measures of impact relate to the epidemiological outcomes observed, including the duration of the impact of each round of IRS and whether these residual effects are underestimated by standard bioefficacy test results. Additionally, during the trial a successful LLIN mass distribution campaign increased the use of at least partially effective standard LLINs to more than 90% without affecting the observed impact of IRS on entomological indicators, suggesting that IRS with PM can provide effective vector control, including in some settings where pyrethroid-only ITNs are widely used.

## Supplementary Information

**Additional file 1: Table S1.** Overall densities and species group distributions for Anopheles mosquitoes collected in CDC LTs (top) and with HLCs (bottom), during the cluster-randomized trial in Mozambique: 2016–2018. All species group designations were based on morphological identifications in the field. **Table S2.** Results of the molecular anlayses. **Table S2a.** Mosquito specimens with a nucleic acid test, by morphological field ID. **Table S2b.** Specimens with a valid molecular species ID. **Table S3.** Monthly trends in estimates of EIRs, in IRS and no-IRS clusters. **Figure S1.** Average (geometric mean) monthly entomological inoculation rate estimates from villages in No IRS (red) and IRS (blue) clusters.

## Data Availability

The datasets analysed here are freely available from the corresponding author upon reasonable request.

## Abbreviations

3GIRs: Third generation indoor residual spray
AIRS: Africa indoor residual spraying
CDC LT: US Centers for Disease Control Light Trap
CI_95_: 95% Confidence interval
EIR: Entomological inoculation rate
IHLC: Human landing collection
RS: Indoor residual spray
LLIN: Long lasting insecticidal net
NMCP: National malaria control programme
PM: Pirimiphos-methyl
PMI: US President’s Malaria Initiative
WHO: World Health Organization
